# Pneumococcal and Influenza Vaccination Coverage in Patients with Heart Failure: A Systematic Review

**DOI:** 10.3390/jcm13113029

**Published:** 2024-05-21

**Authors:** Dimitrios Papagiannis, Christos Kourek, Alexandros Briasoulis, Evangelos C. Fradelos, Evangelia D. Papagianni, Ilias Papadimopoulos, Grigorios Giamouzis, John Skoularigis, Andrew Xanthopoulos

**Affiliations:** 1 Public Health & Adults Immunization Lab, Department of Nursing, School of Health Sciences, University of Thessaly, 41110 Larissa, Greece; dpapajon@gmail.com; 2 Department of Cardiology, 417 Army Share Fund Hospital of Athens (NIMTS), 11521 Athens, Greece; chris.kourek.92@gmail.com; 3 Department of Clinical Therapeutics, Faculty of Medicine, Alexandra Hospital, National and Kapodistrian University of Athens, 11528 Athens, Greece; alexbriasoulis@gmail.com; 4 Laboratory of Clinical Nursing, Department of Nursing, University of Thessaly, 41110 Larissa, Greece; evagelosfradelos@hotmail.com; 5 School of Health Sciences, University of Thessaly, University General Hospital of Larissa, 41500 Larissa, Greece; evelynpapagianni@gmail.com; 6 Alma Mater Studiorum-Medicine and Surgery, University of Bologna, Via Zamboni, 33, 40126 Bologna, Italy; ilias.papadimopoulos@studio.unibo.it; 7 Department of Cardiology, University Hospital of Larissa, 41100 Larissa, Greece; grgiamouzis@gmail.com

**Keywords:** heart failure, vaccination, influenza, pneumococcal disease, vaccination coverage, immune system

## Abstract

**Background/Objectives:** As heart failure (HF) patients face increased vulnerability to respiratory infections, optimizing pneumococcal and influenza vaccination coverage becomes pivotal for mitigating additional health risks and reducing hospitalizations, morbidity, and mortality rates within this population. In this specific subpopulation of patients, vaccination coverage for pneumococcal and influenza holds heightened significance compared to other vaccines due to their susceptibility to respiratory infections, which can exacerbate existing cardiovascular conditions and lead to severe complications or even death. However, despite the recognized benefits, vaccination coverage among HF patients remains below expectations. The aim of the present systematic review was to assess the vaccination coverage for influenza and pneumococcus in HF patients from 2005 to 2023 and the vaccination’s effects on survival and hospitalizations. **Methods:** The authors developed the protocol of the review in accordance with the PRISMA guidelines, and the search was performed in databases including PubMed and Scopus. After the initial search, 851 studies were found in PubMed Library and 1961 in Scopus (total of 2812 studies). **Results:** After the initial evaluation, 23 publications were finally included in the analysis. The total study population consisted of 6,093,497 participants. Regarding the influenza vaccine, vaccination coverage ranged from low rates of 2.5% to very high rates of 97%, while the respective pneumococcal vaccination coverage ranged from 20% to 84.6%. Most studies demonstrated a beneficial effect of vaccination on survival and hospitalizations. **Conclusions:** The present systematic review study showed a wide variety of vaccination coverage among patients with heart failure.

## 1. Introduction

Heart failure (HF) affects more than 6.5 million individuals in the United States, and this number is expected to increase significantly by 2030 [1,2]. HF is the major cause of hospitalization in the elderly and has a significant economic impact on patients, their families, and healthcare systems worldwide [3]. HF is a clinical syndrome frequently characterized by acute exacerbations and onset of symptoms, and vaccination against influenza and pneumococcus in these patients could be offered as a cost-effective intervention to improve quality of life and reduce hospitalizations [4]. The benefits of influenza vaccination coverage as a measure of secondary prevention in ischemic heart disease have been already reported in previous studies [5]. However, evidence for influenza and pneumococcal vaccination coverage, specifically in HF patients, still remains limited.

Influenza infection is a major trigger of cardiac decompensation in patients with HF [6]. Knowledge regarding the impact of influenza vaccination on cardiovascular mortality in these patients is still under investigation. Influenza vaccination is recommended by most National Immunizations Programs and cardiac societies for patients with HF [5,7,8]. People over the age of 65 are at increased risk of getting ill and developing more severe symptoms of pneumococcal disease or influenza, including several complications [9,10]. While studies have shown the benefit of these vaccines in the general public [11,12], research is still ongoing on their usefulness in HF patients. The primary indicator of successful vaccination programs is high vaccination coverage rates among frail patients. The World Health Organization suggests routine annual influenza vaccinations for everyone aged 6 months and older who do not have contraindications to vaccination, and especially for population subgroups who are the most vulnerable to developing serious complications from airborne transmitted infections, including the elderly, people with chronic health problems, pregnant women, and young children.

In HF patients, vaccination coverage for pneumococcal and influenza holds heightened significance compared to other vaccines due to their susceptibility to respiratory infections, which can exacerbate existing cardiovascular conditions and lead to severe complications or even death [9,10]. Pneumococcal and influenza vaccines specifically target pathogens known to cause respiratory illnesses prevalent in this population, thereby reducing the risk of pneumonia, exacerbations of heart failure, and cardiovascular events [4,5]. Ensuring high vaccination rates for these diseases not only safeguards individual health but also helps alleviate the strain on healthcare systems by minimizing hospitalizations and associated costs [11,12]. Thus, prioritizing pneumococcal and influenza vaccination in heart failure patients constitutes a critical preventive measure for preserving both cardiac and overall well-being. However, despite the current recommendations, vaccination coverage for pneumococcal and influenza in patients with HF still remains limited.

The aim of this systematic review was to investigate the existing knowledge regarding influenza and pneumococcal vaccination coverage in patients with HF within the last 18 years and analyze the effect of vaccination on HF outcomes including survival and hospitalization rates.

## 2. Materials and Methods

### 2.1. Search Strategy

The initial search was conducted on the PubMed and Scopus databases by 2 independent reviewers, and included published studies from 2005 to 2023 on influenza and pneumococcal vaccination coverage in patients with HF. This systematic review was conducted in accordance with the proposed reporting items for systematic reviews and meta-analyses (PRISMA) search checklist [13]. We also used the PICOS criteria for the eligibility of articles (Table 1). Search terms that were used included the words “vaccination in heart failure patients”, “vaccination coverage in heart failure patients”, or “heart failure and vaccination”. The total process of articles’ exclusion included three rounds. The first exclusion round was performed by reviewing the title, the second exclusion round by reading the abstract, and the third was based on a reading of the full paper.

### 2.2. Selection Criteria

Seroprevalence studies, experimental studies, descriptive studies, observational studies, or studies that included the health benefits of adult influenza and pneumococcal vaccination in patients with HF were included in our review. We excluded case reports or articles that did not include humans, articles where the intervention was only mentioned in the authors’ recommendations, and articles where the impact of the intervention was not sufficiently described.

### 2.3. Data Extraction

For this evaluation, a specific data extraction form was created. To evaluate the caliber and consistency of data gathering, two reviewers (D.P. and A.X.) independently piloted the extraction procedure. Data regarding vaccination coverage, population characteristics, HF patients, interventions, vaccine type, outcome definition, and vaccination status were extracted.

## 3. Results

**Figure 1:**
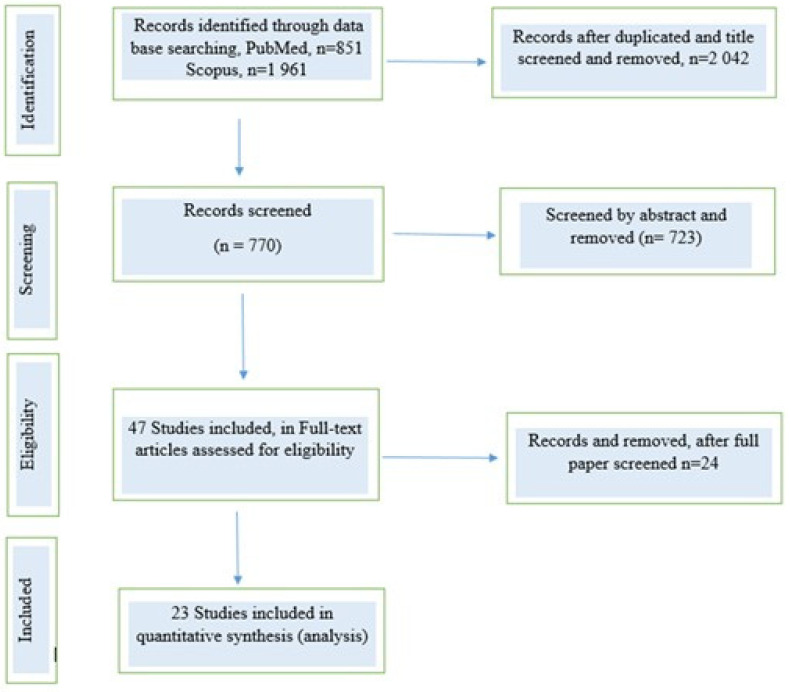
Flowchart of the study screening process in our systematic review.

**Figure 2:**
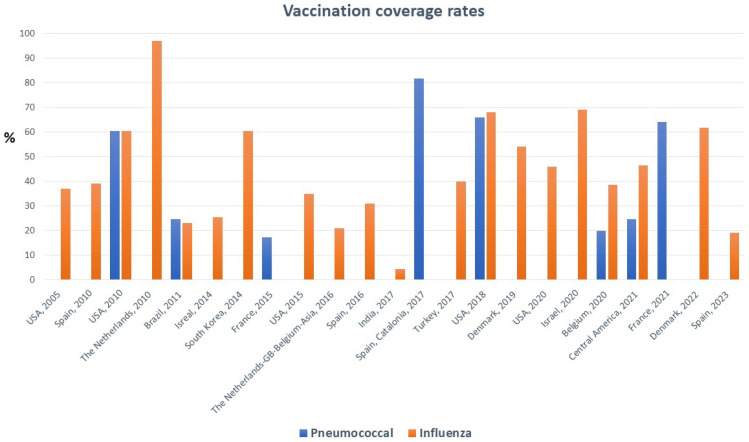
Vaccination coverage rates for pneumococcal and influenza in HF patients in the included studies [14,15,16,17,18,19,20,21,22,23,24,25,26,27,28,29,30,31,32,33,34,35,36]].

The study’s flow chart is demonstrated in Figure 1. In the present systematic review, after the initial screening and the final evaluation of the full texts, we finally included 23 studies. The clinical characteristics of HF patients are demonstrated in Table 2. The results showed variety in the percentages of the vaccination coverage (Table 3). Our results revealed that influenza vaccination coverage ranged from low rates of 2.5% to very high rates of 97% while pneumococcal vaccination coverage ranged from 20% to 84.6% (Figure 2). The coverage of both vaccines was much lower than expected in this special group of HF patients.

The studies’ populations ranged from a small number of 61 participants to 5,102,568 participants and the majority of the studies were performed in Europe. Specifically, ten studies were performed in European countries, five studies in the US, and two in Israel, while the rest were from Brazil, Korea, Turkey, and India.

Ten out of 23 studies [14,15,16,17,18,19,20,21,22,23] investigated the effects of vaccination on HF outcomes including survival, mortality, and hospitalization rates. Most studies demonstrated beneficial effects of vaccination on survival [14,17,18,21,22] and reduced rates of hospitalizations (*p* < 0.05) [15,19,23] (Table 3). A single study showed no association between vaccination and differences in clinical outcomes (*p* > 0.05) [20]. In another study, patients who had been vaccinated had higher rates of ICU admission and need for positive pressure ventilation (*p* < 0.05) [16].

**Table 2:** Patients’ clinical characteristics.

Study	Population	Mean Age (Years) *	Male/Female (%)	Ejection Fraction (%)	NYHA Class (%)	Ischemic Disease (%)	Diabetes (%)	COPD (%)
Examining the coverage of influenza vaccination among people with cardiovascular disease in the United States. Ajani UA et al. [24]	29,991	V (%): 18–49 years 35.9, 50–64 years 26, ≥65 years 38.1 NV (%): 18–49 years 64.2, 50–64 years 24.3, ≥65 years 11.5	V: 44.4/55.6 NV: 49.2/50.8	NA	NA	31.1 with CVD	NA	NA
Vaccination coverage against 2009 seasonal influenza in chronically ill children and adults: analysis of population registries in primary care in Madrid (Spain). Rodríguez-Rieiro C et al. [25]	5,102,568 of which 528,095 had indication for vaccination	Children (%): 6 months to 6 years 45.2, 7–14 years 54.8 Adults (%): 15–29 years 21.3, 30–44 years 33.4, 45–59 years 45.3	In 528,095 cases Children: 58.6/41.4 Adults: 51/49	NA	NA	In 528,095 cases Children: 0.09 Adults: 4.0	In 528,095 cases Children: 0.3 Adults: 14.9	In 528,095 cases Children: 0.5 Adults: 3.4
Prevalence of vaccination rates in systolic heart failure: a prospective study of 549 patients by age, race, ethnicity, and sex in a heart failure disease management program. Hebert K et al. [26]	549	56.41 ± 11.76	69.95/30.05	24.67 ± 9.6	NYHA 1: 23.4 NYHA 2: 30.8 NYHA 3: 28.0 NYHA 4: 17.1	30.4	34	7.9
Improvement of primary care for patients with chronic heart failure: a pilot study. van Lieshout J et al. [27]	77	78 ± 10.3	42/58	NA	NYHA 1: 23 NYHA 2: 41 NYHA 3: 27 NYHA 4: 10	21	31	17
Influenza and pneumococcal vaccination in heart failure: a little applied recommendation. Martins Wde A et al. [28]	61	66.5 ± 11.8	48/52	NA	NA	NA	NA	NA
Influenza vaccine and survival in acute heart failure. Kopel Eran et al. [14]	1964	V: 75.8 ± 9.2 NV: 74.1 ± 10.6	V: 56/44 NV: 55/45	LVEF < 50% V (%): 55 NV (%): 52	V: NYHA I–II 53, NYHA III–IV 47 NV: NYHA I–II 56, NYHA III–IV 44	V: 34 NV: 34	V: 45 NV: 45	NA
Effectiveness of the influenza vaccine at preventing hospitalization due to acute exacerbation of cardiopulmonary disease in Korea from 2011 to 2012. Seo YB et al. [15]	828	67 ± 13	63.9/36.1	NA	NA	58.5	30.9	17.1
Improvement of pneumococcal immunization coverage in older patients. Krypciak S et al. [29]	227	83.6 (79.1–88.7)	44.5/55.5	NA	NA	78 (insufficient cardiac system)	NA	20 (insufficient respiratory system)
The impact of vaccination on influenza-related respiratory failure and mortality in hospitalized elderly patients over the 2013–2014 season. Joshi M et al. [16]	70	V (%): 50–59 years 3, >60 years 97 NV (%): <50 years 3, 50–59 years 25, ≥60 years 72	V: 100/0 NV: 92/8	NA	NA	NA	V: 35 NV: 23	V: 58 NV: 46
Influenza Vaccination in Patients with Chronic Heart Failure: The PARADIGM-HF Trial. Vardeny O et al. [17]	8099	V: 67.9 ± 10.1 NV: 62.7 ± 11.5	V: 80.2/19.8 NV: 77.7/22.3	V: 29.32 ± 6.41 NV: 29.53 ± 6.17	V: NYHA 1: 4.7 NYHA 2: 76.5 NYHA 3: 18.4 NYHA 4: 0.5 NV: NYHA 1: 4.6 NYHA 2: 69.0 NYHA 3: 25.6 NYHA 4: 0.8	V: 61.4 NV: 59.6	V: 40.9 NV: 32.9	NA
Effects of annual influenza vaccination on mortality in patients with heart failure. Blaya-Nováková V et al. [18]	3229	73.6 ± 13.2	37.5/62.5	NA	NA	20.3 (cardiovascular disease)	24.0	63.5 (respiratory disease)
Influenza vaccination in north Indian patients with heart failure. Koul PA et al. [30]	320	V (%): 40–59 years 28.6, ≥60 years 71.4 NV (%): <18 years 1, 18–39 years 8.5, 40–59 years 31.4, ≥60 years 59.2	45.6/54.4	NA	NA	NA	12.2	27.2
Pneumococcal vaccination coverages by age, sex and specific underlying risk conditions among middle-aged and older adults in Catalonia, Spain, 2017. Vila-Córcoles A et al. [31]	2,057,656 of which 63,596 with HF	(%) 50–64 years 50.5, 65–79 years 33.5, ≥80 years 16	46.2/53.8	ΝA	ΝA	5.7 (coronary artery disease)	17	6.4
Influence of influenza vaccination on recurrent hospitalization in patients with heart failure. Kaya H et al. [19]	656	62 ± 13	72/28	32 ± 8	NYHA I–II: 53 NYHA III–IV: 47	51 (coronary artery disease)	22	NA
Vaccination Trends in Patients with Heart Failure: Insights from Get with The Guidelines-Heart Failure. Bhatt AS et al. [20]	136,924 HF patients eligible for influenza vaccination and 256,460 HF patients eligible for pneumococcal vaccination	EIV: 75 (63–84) EPV: 74 (63–84)	EIV: 51.5/48.5 EPV: 51.4/48.6	EIV: 45 (29–58) EPV: 45 (28–58)	NA	NA	EIV: 47.2 EPV: 46.8	EIV: 35.5 EPV: 35.8
Influenza Vaccine in Heart Failure. Modin D et al. [21]	134,048	73.3 ± 13.1	55.9/44.1	NA	NA	39.1	16.0	17.7
Factors influencing the uptake of influenza vaccination in African American patients with heart failure: Findings from a large urban public hospital. Olanipekun T et al. [32]	281	50.5 ± 11.5	57.7/42.3	NA	NA	NA	NA	NA
Influenza Vaccination and Outcome in Heart Failure. Israel Gotsman et al. [22]	6435	76 (66–85)	53/47	HFrEF (%): 28 HFpEF (%): 38 NS (%): 34	NYHA I–II: 63 NYHA III–IV: 37	65	53	21
Vaccination coverage of recommended vaccines and determinants of vaccination in at-risk groups. Boey L et al. [33]	1331 patients of which 200 with HF	71.5 (32–91) in HF	69.5/30.5 in HF	NA	NA	NA	26.3 in total	14.0 in total
Influenza and Pneumococcal Vaccination in Non-Infected Cardio metabolic Patients from the Americas during the COVID-19 Pandemic. A Sub-Analysis of the CorCOVID-LATAM Study. Sosa Liprandi A et al. [34]	4216 of which 538 with HF	60.35 ± 15.39	50.93/49.07	NA	NA	18.24 (coronary artery disease)	21.32	NA
Pneumococcal vaccination coverage in France by general practitioners in adults with a high risk of pneumococcal disease. Kopp A et al. [35]	17,865 of which 673 with HF	75.7 ± 13.5 in HF	48.7/51.3	NA	NA	NA	33.6	61.1
Seasonal influenza vaccine uptake among patients with cardiovascular disease in Denmark, 2017–2019. Christensen DM et al. [36]	1,192,945 of which 185,891 with HF	V: 75 (69–82) NV: 66 (56–75)	V: 56.3/43.7 NV: 59.3/40.7	NA	NA	V: 48.1 NV: 47.2	V: 22.2 NV: 15.6	V: 13.5 NV: 6.4
Effect of Flu Vaccination on Severity and Outcome of Heart Failure Decompensations. Miró O et al. [23]	6147	84 (77–89)	44/56	NA	NYHA I–II: 74.5 NYHA III–IV: 25.5	NA	40.5	22.2

V, vaccinated; NV, not vaccinated; LVEF, left ventricular ejection fraction; EIV, eligible for influenza vaccination; EPV, eligible for pneumococcal vaccination; HFrEF, heart failure with reduced ejection fraction; HFpEF, heart failure with preserved ejection fraction; NS, not specified. Categorial variables are expressed as absolute numbers (population) or percentages. * Continuous variables are expressed either as mean ± SD or median (25th–75th percentiles).

**Table 3:** Influenza and pneumococcal vaccination coverage among studies from 2005 to 2023, and effects on outcomes in HF patients.

	Study	First Author	Year	Population	Country	Flu Vaccination Coverage	Pneumococcal Vaccination Coverage	Effects of Vaccination on HF Outcomes
1	Examining the coverage of influenza vaccination among people with cardiovascular disease in the United States.	Ajani UA [24]	2005	31,044	USA	37.1%		Not investigated
2	Vaccination coverage against 2009 seasonal influenza in chronically ill children and adults: analysis of population registries in primary care in Madrid (Spain).	Rodríguez-Rieiro C [25]	2010	5,102,568	Spain	in patients with diagnosed heart failure coverage reached 39.1%		Not investigated
3	Prevalence of vaccination rates in systolic heart failure: a prospective study of 549 patients by age, race, ethnicity, and sex in a heart failure disease management program.	Hebert K [26]	2010	549	USA	60.5%	60.5%	Not investigated
4	Improvement of primary care for patients with chronic heart failure: a pilot study.	van Lieshout J [27]	2010	77	Netherlands	97%		Not investigated
5	Influenza and pneumococcal vaccination in heart failure: a little applied recommendation.	Martins Wde A [28]	2011	61	Brazil	23.1%	24.6%	Not investigated
6	Influenza vaccine and survival in acute heart failure.	Kopel Eran [14]	2014	1964	Israel	25.5%		The multivariate-adjusted hazard ratios for in-hospital, 1-, and 4-year mortality outcomes of influenza-vaccinated patients were 0.71 (*p* = 0.19), 0.81 (*p* = 0.04), and 0.83 (*p* = 0.006), respectively. Influenza vaccine might improve survival among patients with acute HF.
7	Effectiveness of the influenza vaccine at preventing hospitalization due to acute exacerbation of cardiopulmonary disease in Korea from 2011 to 2012.	Seo YB [15]	2014	828	Korea	54.2% and 60.4%		Conditional logistic regression analysis showed that influenza vaccination significantly reduced the risk of hospitalization, especially due to acute exacerbation of ischemic heart disease and congestive heart failure in patients aged 65 years and older. The estimated vaccine effectiveness in these patients was 56.0% (95% CI 32.1–71.4%, *p* = 0.002). Influenza vaccination was associated with a reduction in the risk of hospitalization due to acute exacerbation of cardiopulmonary disease.
8	Improvement of pneumococcal immunization coverage in older patients.	Krypciak S [29]	2015	227	France		17.2%	Not investigated
9	The impact of vaccination on influenza-related respiratory failure and mortality in hospitalized elderly patients over the 2013–2014 season.	Joshi M [16]	2015	70	USA	35%		Patients who had been vaccinated had higher rates of ICU admission (*p* < 0.05) and need for positive pressure ventilation (*p* < 0.05). There was no protective effect from prior vaccination in preventing hospital admission, respiratory failure, and mortality in this population of older men admitted to the hospital with influenza.
10	Influenza Vaccination in Patients with Chronic Heart Failure: The PARADIGM-HF Trial.	Vardeny O [17]	2016	8099	Netherlands (77.5%), Great Britain (77.2%), and Belgium (67.5%), Asia (2.6%)	21%		Influenza vaccination was associated with a reduced risk for all-cause mortality in propensity-adjusted (hazard ratio: 0.81; 95% confidence interval: 0.67 to 0.97; *p =* 0.015) models. Vaccination was associated with reduced risk for death.
11	Effects of annual influenza vaccination on mortality in patients with heart failure.	Blaya-Nováková V [18]	2016	3229	Spain	31%		Influenza vaccine was associated with a decreased risk of death during the influenza season (hazard ratio = 0.59, 95% CI = 0.41–0.84), but no protective effect was observed before or after the influenza season.
12	Influenza vaccination in north Indian patients with heart failure.	Koul PA [30]	2017	320	India	4.4%		Not investigated
13	Pneumococcal vaccination coverages by age, sex and specific underlying risk conditions among middle-aged and older adults in Catalonia, Spain, 2017.	Vila-Córcoles A [31]	2017	63,596	Catalonia Spain		81.6%	Not investigated
14	Influence of influenza vaccination on recurrent hospitalization in patients with heart failure.	Kaya H [19]	2017	656	Turkey	40%		Regular influenza vaccination does not influence cardiovascular deaths (16 vs. 19%, *p* = 0.37); however, it decreases heart failure-related hospitalizations (43 vs. 92%, *p* < 0.001) including recurrent episodes of heart failure-related hospitalizations in outpatients with heart failure with reduced ejection fraction (16 vs. 66%, *p* < 0.001).
15	Vaccination Trends in Patients with Heart Failure: Insights from Get with The Guidelines-Heart Failure.	Bhatt AS [20]	2018	313,761	USA	was 68% overall and declined from 70% in 2012 to 2013 to 66% in 2016 to 2017	was 66% overall and decreased over the study period from 71% in 2013 to 60% in 2016	Vaccinated patients had similar rates of 1-year all-cause mortality (adjusted hazard ratio: 0.96 [95% CI: 0.89 to 1.03] for influenza vaccination; adjusted hazard ratio: 0.95 [95% CI: 0.89 to 1.01] for pneumococcal vaccination) compared with those not vaccinated. Vaccination status was not associated with differences in clinical outcomes.
16	Influenza Vaccine in Heart Failure.	Modin D [21]	2019	134,048	Denmark	16% to 54%		Receiving ≥1 vaccinations was associated with an 18% reduced risk of death (all-cause: hazard ratio, 0.82; 95% CI, 0.81–0.84; *p* < 0.001; cardiovascular causes: hazard ratio, 0.82; 95% CI, 0.81–0.84; *p* < 0.001). Influenza vaccination was associated with a reduced risk of both all-cause and cardiovascular death.
17	Factors influencing the uptake of influenza vaccination in African American patients with heart failure: Findings from a large urban public hospital.	Olanipekun T [32]	2020	281	USA	46%		Not investigated
18	Influenza Vaccination and Outcome in Heart Failure.	Israel Gotsman [22]	2020	6435	Israel	69% of the HF cohort		Vaccination was associated with reduced mortality (hazard ratio [HR] 0.77, 95% confidence interval [CI] 0.65 to 0.91, *p* < 0.01) as well as reduced death and cardiovascular hospitalizations (HR 0.83 95% CI 0.76 to 0.90, *p* < 0.001). Influenza vaccination in patients with HF was associated with improved clinical outcome including improved survival and reduced death and hospitalizations.
19	Vaccination coverage of recommended vaccines and determinants of vaccination in at-risk groups.	Boey L [33]	2020	200	Belgium	38.5%	20%	Not investigated
20	Influenza and Pneumococcal Vaccination in Non-Infected Cardio metabolic Patients from the Americas during the COVID-19 Pandemic. A Sub-Analysis of the CorCOVID-LATAM Study.	Sosa Liprandi A [34]	2021	4216	Central America	46.5%	24.6%	Not investigated
21	Pneumococcal vaccination coverage in France by general practitioners in adults with a high risk of pneumococcal disease.	Kopp A [35]	2021	17,865	France		(PPSV23): 18 (64%) in the study and 8 (80%) in the control periods, respectively.	Not investigated
22	Seasonal influenza vaccine uptake among patients with cardiovascular disease in Denmark, 2017–2019.	Christensen DM [36]	2022	397,346	Denmark	61.8%		Not investigated
23	Effect of Flu Vaccination on Severity and Outcome of Heart Failure Decompensations.	Miró O [23]	2023	6147	Spain	19%		Infection triggering decompensation was more common in vaccinated patients (50% vs. 41%; *p* < 0.001). Vaccinated decompensated patients with HF had decreased odds for hospitalization (OR = 0.823, 95% CI = 0.709–0.955). In patients with HF, seasonal flu vaccination is associated with less severe decompensations.

## 4. Discussion

There are sparse data regarding flu and pneumococcal vaccination rates in patients with HF globally. In the present systematic review, we demonstrated the low rates of vaccination coverage in patients with HF, derived from 23 studies, within the time period from 2005 to 2023. The goal of high vaccination coverage for influenza and pneumococcus is to increase overall life expectancy in frail patients and to reduce morbidity, mortality, and hospitalizations.

Recent sub-analyses of big clinical trials report a high incidence of pneumococcal infections in patients with HF, with the incidence rates reaching up to 39 per 1000 patient-years [37]. Moreover, respiratory infections including pneumonia are associated with higher in-hospital mortality (odds ratio, 1.60) [38] and increased rates of acute HF hospitalizations up to 24% [39]. Except for mortality and hospitalizations, patients with HF who are infected by influenza or pneumococcus frequently present with worse clinical symptoms. In a large retrospective study in the US that included 8,189,119 hospitalized HF patients, it was shown that they were at higher risk for worse outcomes such as acute respiratory failure and acute kidney injury [40].

The beneficial effects of influenza and pneumococcal vaccination on the severity of HF and clinical outcomes in patients with HF have been shown in recent studies [20]. The PARADIGM-HF trial showed that patients with HF who were vaccinated against influenza had a significant decrease in the relative risk of all-cause mortality (HR, 0.81; 95% CI, 0.67 to 0.97; *p* = 0.015) [17]. In a meta-analysis by Udell et al. [41], influenza-vaccinated patients had a lower risk for a composite of major cardiovascular events compared to the unvaccinated patients (2.9% vs. 4.7%; relative risk 0.64, 95% CI, 0.48–0.86, *p* = 0.003), with the effect of vaccination being greater in patients with higher-risk coronary disease. In another randomized double-blind placebo-controlled trial, influenza vaccination administered shortly after myocardial infarction reduced the rate of the composite primary endpoint (all-cause death, myocardial infarction, or stent thrombosis) (HR 0.72, 95% CI 0.52–0.99, *p* = 0.040), the rates of all-cause death (HR 0.59, 95% CI 0.39–0.89, *p* = 0.010), and the rates of cardiovascular death (HR 0.59, 95% CI 0.39–0.90, *p* = 0.014) at 12 months compared to the placebo group [42]. Another finding observed in our review was that vaccination against influenza before hospital admission was associated with a significant decrease in risk of all-cause mortality and cardiovascular hospitalizations in patients hospitalized with acute HF. In general, 10 out of the 23 studies [14,15,16,17,18,19,20,21,22,23] in our systematic review investigated the effects of vaccination on HF outcomes, including survival, mortality, and hospitalization rates. Influenza vaccination coverage was associated with lower 1-year, 4-year, and all-cause mortality rates [14,17,18,21,22] and reduced rates of hospitalizations due to HF symptoms [15,19,23]. Only a single study showed no association between vaccination and differences in clinical outcomes [20]. Unfortunately, there were no studies assessing the effect of pneumococcal vaccination on HF outcomes. On the contrary, there was a single study where patients who had been vaccinated presented with higher rates of ICU admission and the need for positive pressure ventilation [16]. The reason may be the fact that the sample size consisted of hospitalized elderly patients with multiple comorbidities of high severity including COPD, congestive HF, and cancer who had poor prognoses.

On the other hand, pneumococcal disease is associated with significant morbidity and mortality in both developing and developed countries, resulting in 1.6 million deaths annually. By 2040, the burden of pneumococcal disease among the elderly is expected to be double [43]. Pneumococcal vaccination in HF is also quite significant, as shown by clinical trials. Specifically, a recent meta-analysis that included 18 studies with 716,108 participants demonstrated the protective effect of 23-valent polysaccharide pneumococcal vaccination (PPV23) in patients with cardiovascular events by reducing the rates of cardiovascular events (RR 0.91; 95% CI 0.84–0.99) and myocardial infarction (RR 0.88; 95% CI 0.79–0.98), as well as the risk of all-cause mortality, in all age groups (RR 0.78; 95% CI 0.68–0.88) [44]. This effect seemed to be higher in patients aged ≥65 years. Finally, Jaiswal et al. showed that pneumococcal vaccination was associated with a decrease in the incidence of myocardial infarction (HR, 0.73 (95% CI: 0.56−0.96), *p* = 0.02), without significant reduction in CV mortality (HR, 0.87 (95% CI: 0.72−1.07), *p* = 0.18) and stroke (HR, 1.01 (95% CI: 0.93−1.10), *p* = 0.82) [45]. Pneumococcal vaccination is associated with a decreased risk of all-cause mortality in patients at very high cardiovascular risk [46]. The CDC and ECDC recommend immunization with polysaccharide and conjugate vaccines against pneumococcal infection for older adults, individuals at risk, and high-risk patients with HF [47,48,49]. The protective efficacy of polysaccharide vaccines has been consistently shown in the literature [50,51,52]. Systematic reviews and meta-analyses support the effectiveness of conjugate vaccines against pneumococcal disease in adults [53,54].

Another virus infection, the COVID-19 pandemic, caused by the severe acute respiratory syndrome coronavirus 2 (SARS-CoV-2), has significantly affected public health on a global scale. COVID-19 has been associated with the development of HF via myocardial infarction, myocarditis, microthrombi, and stress cardiomyopathy [55]. HF is a common condition among patients with COVID-19, estimated at between 4.9% and 13% of hospitalized patients with COVID-19, leading to poor prognoses [56,57]. Johnson et al. performed a study during the pandemic in order to assess the benefits of vaccination against COVID-19 in patients with HF [58]. Immunization against COVID-19 was shown to decrease all-cause hospitalizations and death rates in high-risk patients with HF [58].

Clinical applications of the immunization protocols are not limited to the population with HF. The improvement of general health practices and cardiovascular outpatient programs could significantly increase immunization rates among patients with comorbidities. National vaccination programs for the elderly and other special populations are intricate, with socioeconomic and income differences. There are opportunities to expand and improve national immunization programs. The recent COVID-19 pandemic demonstrated the importance of technology and telemedicine, such as vaccine registration systems, in order to support public immunization programs [59]. To ensure that HF patients receive the best possible immunization coverage, public health authorities should concentrate their efforts on yearly vaccination campaigns.

Health campaigns need to focus on appropriate planning, easy implementation, and continuous evaluation, which are crucial factors for their success [60]. Despite there being significant knowledge about the benefits of vaccinations [61], there are also differences in vaccination programs for adults around the world [62]. In the present review, we presented the results of vaccination coverage in HF patients, including rates; however, the rates remain lower than suggested. Influenza vaccination coverage is frequently lower than the World Health Organization (WHO) and European Union (EU) target of 75% of older adults [63]. The percentage of pneumococcal vaccination varies in older adults. National guidelines and immunization programs differ, recommending either the polysaccharide vaccine or the conjugate vaccine, or even both. Streptococcus pneumonia remains a major cause of morbidity and mortality in high-risk groups and elderly people, and as a result, recommendations focus on routine vaccination against pneumococcal diseases in these individuals [64]. The global target for vaccination coverage against pneumococcus seems to be far from the proposed target of 60% for high-risk adults aged <65 years and 90% for elderly people [31,65]. The present review highlighted the difficulties in comparing pneumococcal vaccination coverages in distinct areas due to the different characteristics of national healthcare systems and vaccination programs. We also reported low percentages of immunization against influenza and pneumococcus during the COVID-19 pandemic, as well as major geographic differences in vaccination rates among them. Most likely, the pandemic played a crucial role in the reduction of routine vaccination coverage. It would be quite interesting to perform a sub-analysis of the vaccination coverage rates before and after the COVID-19 period in order to assess its impact on vaccination, as the present review was also a meta-analysis. Comparing vaccination rates in the same countries before and after the pandemic, we observed that in most places, such as the USA, Israel, France, and Denmark, the vaccination rates increased during and after the pandemic, while in other countries such as Spain and Belgium, the rates decreased. However, the sample sizes in the same country are quite different before and after the pandemic, and thus, we cannot extract safe conclusions.

Finally, another finding of the systematic review was that immigrants were less likely to be vaccinated compared to native-born people. Refugees and immigrant groups presented with low immunization rates, putting them at higher risk for infectious diseases which could be prevented by vaccination [66]. The fact that the study sample may include high percentages of immigrants and refugees could explain the reason why some studies in our systematic review presented very low vaccination coverage rates amongst HF patients. Furthermore, in developing countries in Africa and Asia, as well as countries with a very large population and low socioeconomic status, people with cardiovascular diseases do not have the opportunity to vaccinate against pneumococcal and influenza, especially when there is not a 100% compensation rate for vaccines and the economic status of the country remains low. As a result, an inadequate vaccine supply is being observed. In these countries, there is a lack of awareness, as educational campaigns about the beneficial effects of vaccination are quite limited. Challenges related to vaccine accessibility, such as geographic barriers, limited healthcare professionals, or transportation issues, may also hinder HF patients’ ability to receive vaccination coverage. Finally, the national health policy of each country may be another significant factor for differences in vaccination coverage among countries. Even within the same country, the extensive time range could affect vaccination, as it may have changed several times from 2005 until 2023.

### Limitations and Future Directions

The present review has several limitations. Most of the studies were considered as being at a high risk of bias, mainly due to the lack of control for the confounders. Specifically, information bias in observational studies, particularly in those with questionnaires about vaccination coverage, should be taken into consideration. Moreover, due to the heterogeneity of the studies, were could not generalize and compare the results from different studies, nor perform a meta-analysis. Another significant limitation is the fact that the extensive time range brings a bias in interpreting the results. Specifically, within the same country, the national health policy regarding vaccination may have changed several times within this 18-year time period. In addition, the economic level of each country may be an important factor for vaccination coverage, as several countries have a 100% compensation rate for vaccines while others have a price for them, and as a result, many HF patients cannot afford them. Finally, protection from pneumococcal and influenza vaccination in the COVID-19 era may include bias, as a great number of HF patients vaccinated for pneumococcal and influenza infection could have presented with an episode of decompensated HF due to COVID-19 infection.

Future directions of pneumococcal and influenza vaccination coverage in patients with cardiovascular diseases, and especially HF, should involve the implementation of targeted campaigns in order to raise awareness among both patients and healthcare providers about the importance of vaccination in managing HF and reducing complications, as well as collaboration between primary care, public health sectors, and the hospital setting which may be pivotal in establishing comprehensive vaccination programs tailored to the unique needs of HF patients. Finally, ongoing research into vaccine effectiveness and the development of novel delivery methods could offer promising avenues for optimizing protection and reducing disease burden in this population.

## 5. Conclusions

Patients with heart failure are vulnerable to virus infections and vaccination against influenza and pneumococcus is necessary to reduce mortality and morbidity. High vaccination coverage in patients with comorbidities is the only way to achieve the desired results. Our systematic review demonstrated a variety of influenza and pneumococcal vaccination coverage rates among patients with HF. In national health systems, further effort is necessary in order to develop effective immunization programs and increase vaccination coverage in these patients.

## Figures and Tables

**Table 1:** PICOS criteria for eligibility of articles.

PICOS	Description
Population	Vaccinated patients with heart failure as a “risk group” (as defined by the authors of the paper)
Interventions	Vaccination with flu vaccine or PCV or PPV23
Comparator	Comparators as defined by the authors of the study, can include: No vaccination Placebo Vaccination with another product (tetanus, diphtheria) Co-administration of pneumococcal vaccine with influenza vaccine compared with vaccination with only one, or compared with no vaccination Pneumococcal vaccination of a subgroup compared with another subgroup (e.g., Heart failure patients compared with healthy adults)
Outcomes	Health benefits of heart failure patients and flu and pneumococcal vaccination, including: Healthcare cost savings Reduction of mortality Care-related productivity gains Prevention and amelioration of comorbidities Reduction in hospital infections
Study Design	Seroprevalence studies, experimental, descriptive, observational, or studies that included health benefits of adult influenza and pneumococcal vaccination in patients with heart failure.

## Data Availability

No new data were created.
